# Geographic Differences in Genetic Locus Linkages for *Borrelia burgdorferi*

**DOI:** 10.3201/eid1607.091452

**Published:** 2010-07

**Authors:** Bridgit Travinsky, Jonas Bunikis, Alan G. Barbour

**Affiliations:** Author affiliation: University of California, Irvine, California, USA

**Keywords:** Lyme disease, Lyme borreliosis, Ixodes scapularis, Ixodes pacificus, Borrelia burgdorferi, genotype, ospC, bacteria, vector-borne infections, dispatch

## Abstract

*Borrelia burdorferi* genotype in the northeastern United States is associated with Lyme borreliosis severity. Analysis of DNA sequences of the outer surface protein C gene and *rrs-rrlA* intergenic spacer from extracts of *Ixodes* spp. ticks in 3 US regions showed linkage disequilibrium between the 2 loci within a region but not consistently between regions.

Most bacterial pathogens comprise a variety of strains in various proportions. For *Borrelia burgdorferi*, an agent of Lyme borreliosis, strains differ in their reservoir host preferences (*1*), propensities to disseminate in humans (*2**,**3*), and prevalences in ticks by geographic area (*4**,**5*). Strain identification of *B. burgdorferi* now is predominantly based on DNA sequences of either of 2 genetic loci: 1) the plasmid-borne, highly polymorphic *ospC* gene, which encodes outer surface protein C (*6**,**7*), or 2) the intergenic spacer (IGS) between the *rrs* and *rrlA* rDNA, here called IGS1. Other loci for genotyping are the plasmid-borne *ospA* gene (*7*) and the rrfA-rrlB rDNA intergenic spacer, here called IGS2 (*8*). The apparent clonality of *B. burgdorferi* was justification for inferring strain identity from a single locus (*9**,**10*), but the extent of genomewide genetic exchange in this species may have been underestimated (*6*).

Given reports of an association between disease severity and *B. burgdorferi* genotype (*2**,**3*), prediction of a strain’s virulence potential from its genotype has clinical, diagnostic, and epidemiologic relevance. But is a single locus sufficient for this assessment?

## The Study

To investigate this issue, we determined sequences of *ospC* and IGS1 loci, and in selected cases the *ospA* and IGS2 loci, in 1,522 DNA extracts from *B. burgdorferi*–infected *Ixodes scapularis* nymphs collected from the northeastern, mid-Atlantic, and north-central United States during the summers of 2004, 2005, 2006, and 2007, as described (*4**,**11*). We also included results from 214 infected *I. pacificus* nymphs collected in Mendocino County, California (*5*); 20 infected *I. pacificus* adults from Contra Costa County, California (J. Bunikis and A.G. Barbour, unpub. data); and 10 *B. burgdorferi* genomes (strains B31, ZS7, 156a, 64b, 72a, 118a, WI91-23, 94a, 29805, and CA-11.2a), for which sequences are publicly available (www.ncbi.nlm.nih.gov). Multilocus sequence typing (MLST), based on 8 chromosomal housekeeping genes, had been carried out for several strains represented in the extracts (Table) (*4**,**12*). The corresponding MLST types of the 10 genome sequences were assigned by reference to a *B. burgdorferi* MLST database (http://borrelia.mlst.net) (*12*). For this study, we also determined the MLST type of strain CA8.

The methods for 1) DNA extraction from ticks (*11*), 2) PCR amplification of *ospC*, *ospA*, and IGS1 (*7*), 3) amplification of IGS2 (*8*), and 4) amplification of 8 chromosomal loci for MLST (*12*) have been described. Sequences for both strands were determined from either PCR products or cloned fragments with custom primers (*7*). We followed the basic nomenclature of Wang et al. (*13*) until, after exhausting the alphabet, we assigned both a letter and, arbitrarily, the number 3 (e.g., C3) when a new nucleotide sequence differed by >8% from known *ospC* alleles. We distinguished *ospC* variants with <1% sequence difference by adding a lowercase letter, e.g., Da and Db. Except for *ospC* D3 and Oa, novel polymorphisms were confirmed in at least 1 other sample. To simplify IGS1 nomenclature, we numbered types sequentially, beginning with the original 9 types (*7*); *ospA* alleles (*7*) and IGS2 loci were likewise sequentially numbered. The Table A1 provides accession numbers for all sequences, as well as original and revised names for IGS1 sequences.

For 741 *Ixodes* ticks from northeastern and north-central United States or from northern California, 1 *ospC* allele was identified and sequenced. In the remaining samples, we found a mixture of strains or evidence of >2 *ospC* and/or >2 IGS sequences (*9*). In 678 (91%) of the 741 samples with a single *ospC*, the allele could be matched with particular IGS1 (Table). We identified 9 unique *ospC* sequences: Fc, Ob, Ub, A3, B3, C3, D3, E3, and F3, all from the north-central United States. Alleles H3 and I3 of California were recently reported by Girard et al. (*5*). Of 32 codon-aligned *ospC* sequences, 6 pairs and 1 trio (Fa, Fb, and Fc) differed in sequence by <1% (Figure, panel A). Nine novel IGS1 sequences, numbered 24–31 and 33, were discovered in samples from which *ospC* alleles were determined.

**Figure:**
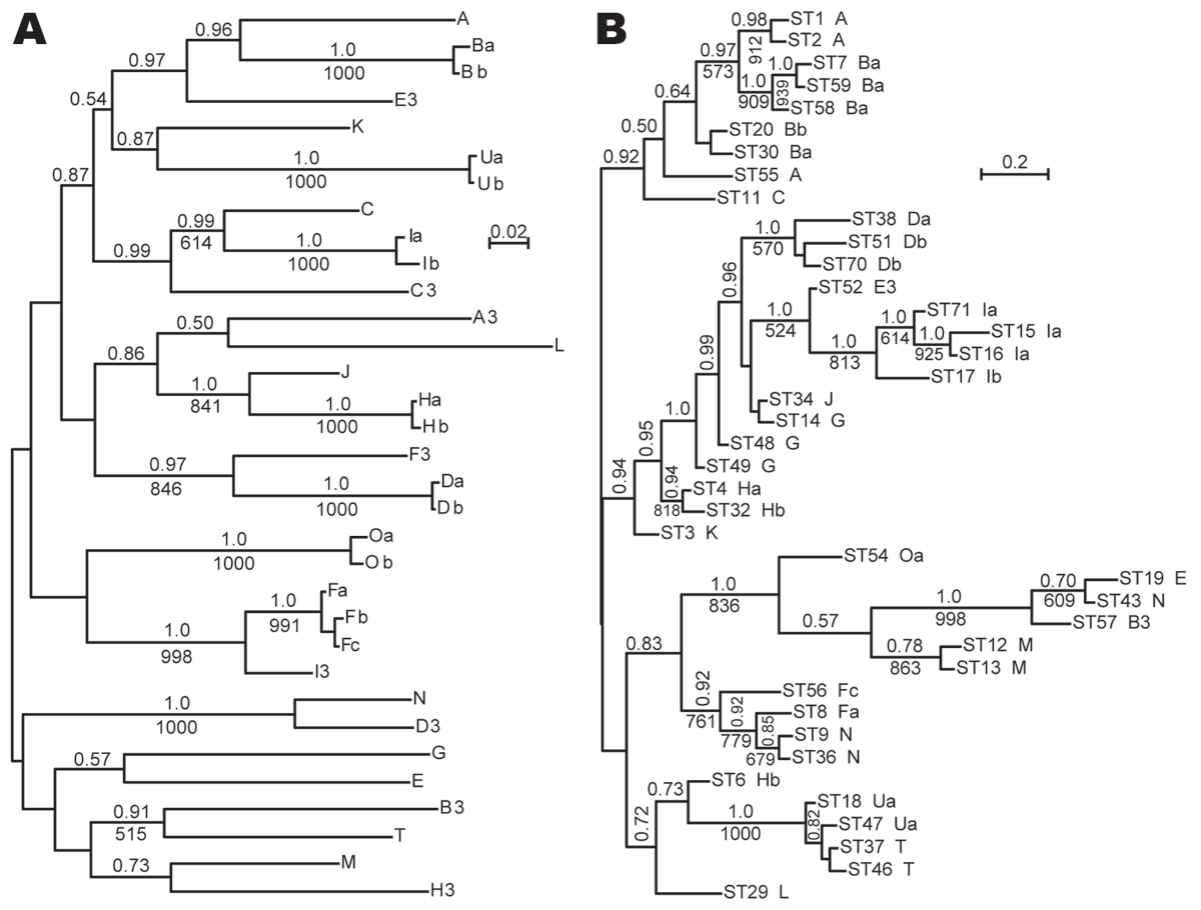
A) Bayesian and maximum-likelihood phylogenetic inference of outer surface protein C (*ospC*) gene sequences and B) concatenated multilocus sequence typing (MLST) sequences of *Borrelia burgdorferi*. Sequences were aligned by codon. Labels at the tips refer to *ospC* alleles (A) or MLST (ST) and linked *ospC* alleles (B; Table). Consensus phylograms were the output of the MrBayes version 3.1.2 algorithm (http://mrbayes.csit.fsu.edu). There were 500,000 generations with the first 1,000 discarded. Nodes with posterior probabilities of >0.5 are indicated by values above the branches. Below the branches are integer values for nodes with support of >500 of 1,000 bootstrap iterations of the maximum-likelihood method, as carried out with the PhyML 3.0 algorithm (www.atgc-montpellier.fr/phyml). For both data sets and both algorithms, the models were general time reversible with empirical estimations of the proportions of invariant sites and gamma shape parameters. Scale bars indicate genetic distance. GenBank accession numbers for sequences are given in the Table A1.

When we confined analysis to samples from northeastern states, we confirmed linkage disequilibrium between *ospC* and IGS1 loci (*7**,**10**,**14*). However, when results from north-central states and California were included, a different picture emerged (Table, Figure, panel B). Most of the *ospC* alleles showed concordance with the chromosomal loci; monophyletic MLST showed either the same *ospC* allele or a minor variant of it. However, in several instances, the *ospC* alleles were linked to different IGS1 sequences, different *ospA* sequences, and/or different MLST with internal nodes in common. We observed this linkage for *ospC* alleles A, G, Hb, and N. In the case of *ospC* Hb, the shared internal node was deep.

We applied the Simpson index of diversity, as implemented by Hunter and Gaston (*15*), to the data in the Table to compare the discriminatory power (DP) of genotyping on the basis of a combination of *ospC* and IGS1 sequences with genotyping by 8-locus MLST (*12*). For double-locus typing, there were 43 types were found for 678 strains; DP value was 0.96. For MLST in this data set, 36 types were shown for 554 strains; DP was 0.95. In the study of Hoen et al. in which selection was made for geographic isolation, 37 types were distributed among 78 strains; DP was 0.97 (*4*).

## Conclusions

Dependence on a single locus for typing may falsely identify different lineages as the same, especially when the samples come from different regions. Other loci may be as informative as *ospC* or IGS1, but the abundance of extant sequences for these loci justifies their continued use. Uncertainties about the linkage of *ospC* and IGS1 usually can be resolved by sequencing the *ospA* allele (Table). IGS2 provided little additional information in this study.

One interpretation of these findings is that lateral gene transfer of all or nearly all of an *ospC* gene has occurred between different genetic lineages. We previously had not detected recombination at the IGS1 locus on the chromosome (*7*), but there may be recombination at other chromosomal loci, as well as plasmid loci (*6*). Besides extending the understanding of the geographic structuring of the *B. burgdorferi* population, the results indicate that the *ospC* allele does not fully represent the complexity of *B. burgdorferi* lineages; thus, inferring phenotypes on the basis of this single locus should be made with caution.

## Appendix

**Table A1:** GenBank accession numbers of sequences of *Borrelia burgdorferi* in this study*

Strain name	*ospC* allele	*ospC* accession no.	Former IGS1† name	Revised IGS1 name	IGS1 accession no.	*ospA* allele	*ospA* accession no.	IGS2‡ name	IGS2 accession no.
B31	A	AE000792	1A	1	AE000783	1	AE000790	1	**GQ463603**
CA4	A	EU377746	1A-684	10	EU377801	23	**GQ443123**		
CA6	A	EU377748	1A-684	10	EU377803				
2206617	A	AE000792	1A-684/672	11	**GQ478289**	22	**GQ443122**		
64b	Ba	CP001422	3A	3	ABKA02000001	3	CP001421		
B373	Ba	EU377779	3B	3	EU377795				
51405UT	Ba	EU375825	6A	6	EU375815	14	**GQ443114**		
ZS7	Bb	NC_011724	3D	16	NC_011728	28	CP001199		
JD1	Ca	DQ437462	5G	24	DQ437478				
BL515	Ca	EU377774	5G	24	EU377790				
OC4	Da	AF029863	5A	5	AY275201				
516113	Da	AY275217	5A	5	AY275201	5	**GQ433636**	4	**GQ463606**
424404	Db	**GQ478283**	5A	5	AY275201	18	**GQ443118**	7	**GQ463609**
CA-11.2a	Db	CP001484	5A-239	19	ABJY02000007	27	CP001473		
N40	E	AY275221	9A	9	AY275211	9	M57248		
B348	E	AF467875	9C	9	AF467863				
990503	Fa	AY275225	4C	17	**GQ130198**				
B156	Fa	EU377776	4C	17	EU377792				
MI407	Fb	EF537433	4D	18	EF537367				
1469205	Fc	**GQ478285**	4D	18	EF537367	13	**GQ443113**	6	**GQ463608**
MR616	G	EU377771	6B	26	EU377787				
72a	G	CP001375	6B	26	ABGJ02000006	9	CP001370		
1468503	G	AY275223	5C	22	**GQ130201**	21	**GQ443121**		
B509	Ha	EU377781	2D	12	EU377797				
156a	Hb	CP001271	2D	12	ABCV02000001	2	CP001257		
519014UT	Hb	EU375831	2D	12	EU375823				
519512	Hb	**GQ478286**	2D	12	EU375823				
CA92-0953	Hb	EU377751	2D-713	13	EU377806				
B500	Ia	AF467878	7A	7	AF467866				
B331	Ia	AF467874	7A	7	AF467862	7	**GQ443107**		
1472505	Ia	AY275219	7A	7	AY275205	10	**GQ443110**		
WI91-23	Ia	CP001446	7A	7	ABJW02000006	11	CP001447		
CA92-1096	Ib	EU377752	7A	7	EU377807				
CA337	Ib	EU377752	7A	7	EU377807	30	GU815347		
118a	J	CP001535	5B	20	ABGI02000001	8	CP001542		
297	K	AY275214	2B	2	AY275192	2	X85442	2	**GQ463604**
501604	K	AY275214	2A	2	AY275191				
149901	K	AY275214	2E	14	**GQ120104**	31	GU815348		
47703UT	L	EU375832	2E	14	**GQ120104**				
29805	M	CP001550	6A	6	ABJX02000028	2	CP001554	3	**GQ463605**
CA92-1337	M	EU377753	6A	6	EU377808				
MR661	N	EU377775	4A	4	EU377791	4	**GQ433635**		
500203	N	AY275216	4A	4	AY275199				
MI418	N	EF537430	5E	23	EF537363				
51108	N	AY275216	5E	23	**GQ130203**				
501427	Oa	**FJ997281**	6C	27	AY275204				
2207807	Ob	**FJ997282**	6A	6	ABJX02000028				
23509	T	AY275222	8C	28	AY275209				
1476702	T	AY275222	8C-808	29	**GQ478288**	20	**GQ443120**		
94a	Ua	CP001493	8A	8	ABGK02000002	8	CP001500		
B485	Ua	EU377769	8A	8	EU377785				
48802	Ua	AY275220	8A	8	ABGK02000002	16	**GQ443116**		
2207116	Ua	EU377769	8A	8	EU377785	12	**GQ443112**	10	**GQ463612**
426905	Ub	**GQ478287**	8E	30	**GQ130197**	8	**GQ443108**	9	**GQ463611**
2206613	A3	**EF592541**	2E	14	**GQ120104**	19	**GQ443119**		
2250201	B3	**EF592542**	5E	23	**GQ130203**	17	**GQ443117**		
50202	C3	**EF592543**	4C	17	**GQ130198**	15	**GQ443115**	5	**GQ463607**
2150902	D3	**EF592544**	New	31	**GQ478290**				
2127701	E3	**EF592545**	5B	20	**GQ130200**			8	**GQ463610**
HRT25	E3	**EF592545**	5A-725	21	EU886975	24	**GQ443124**		
LMR28	E3	**EF592545**	5A	5	AY275201	25	**GQ443125**		
1456802	F3	**EF592547**	5A	5	AY275201				
CA8	H3	FJ932733	5A8	25	EU886974	26	**GQ247743**		
CA11	I3	FJ932734	4C	17	**GQ130198**				
CA12	I3	FJ932734	4C	17	**GQ130198**				

***Boldface** indicates new accession number from this study. †IGS1, *rrs-rrlA* intergenic spacer region. ‡IGS2, *rrf-rrlB* intergenic spacer.

**Table:** Linkages between ospC alleles and other loci in *Borrelia burgdorferi* strains*

*ospC*	IGS1	Geographic region*	Representative cultured isolate or tick sample†	IGS1-*ospC* associations‡	*ospA*	IGS2	MLST§
A	1	1, 2	**B31**	45/52	1	1	1
A	11	2	*2206617*	4/4	22	1	55
A	10	3	CA4, CA6	14/18	23	1	2
Ba	3	1	**64b**, B373	39/41	3	1	7,58,59
Ba	6	2	51405UT	7/9	14	1	30
Bb	16	4	**ZS7**	–	28	–	20
C	24	1	JD1, BL515	10/10	8	5	11
Da	5	1	*516113*	13/14	5	4	38
Db	5	2	*424404*	13/15	18	7	51
Db	19	3	**CA11.2A**	16/16	27	4	70
E	9	1, 2	N40, B348	17/19	9	1	19
Fa	17	1, 2, 3	B156	61/64	3	4	8
Fb	18	2	MI407	14/19	8	6	–
Fc	18	2	*1469205*	7/8	13	6	56
G	26	1	**72a**, MR616	10/11	9	4	14
G	22	2, 3	*1468503*	9/10	21	4	48,49
Ha/Hb	12	1	B509/**156a**	13/13	2	2	4
Hb	12	2	519014UT	56/65	11	2	32
Hb	13	3	CA92-0953	20/20	23	2	6
Ia	7	1	B500, B331	12/16	7	4	15,16
Ia	7	2	**WI91-23**	5/5	11	4	71
Ib	7	3	CA92-1096	–	30	4	17
J	20	1, 2	**118a**	3/5	8	4	34
K	2	1	297	67/68	2	2	3
K	14	2	*149901*	7/10	31	2	–
L	14	2	47703UT	23/25	8	2	29
M	6	1	**29805**	4/4	2	3	12
M	6	2, 3	CA92-1337	16/16	17	3	13
N	4	1	MR661, *500203*	41/41	4	10	9,36
N	23	2	*51108*	8/10	2	1	43
Oa	27	1	*501427*	1/1	–	–	54
Ob	6	2	*2207807*	6/7	2	–	–
T	28	1	23509	16/16	8	4	37
T	29	2	1476702	10/11	20	4	46
Ua	8	1	**94a**, B485	19/19	8	4	18
Ua	8	2	*48802*	4/4	16	4	47
Ua	17	2	*2207116*	4/4	12	10	–
Ub	30	2	*426905*	3/3	8	9	–
A3	14	2	*2206613*	6/6	19	2	–
B3	23	1, 2	*2250201*	3/3	17	1	57
C3	17	2	*50202*	6/9	15	5	–
D3	31	2	*2150902*	1/1	–	–	–
E3	20	2	*2127701*	4/4	8	8	52
E3	21	3	HRT25	12/12	24	–	–
E3	5	3	LMR28	12/12	25	–	–
F3	5	2	*1456802*	8/12	8	4	–
H3	25	3	CA8	37/40	26	4	(72)
I3	17	3	CA11, CA12	5/5	27	4	–

*Regions: 1, northeastern United States; 2, north-central United States; 3, northern California; 4, western Europe; *osp*, outer surface protein; IGS, intergenic spacer; MLST, multilocus sequence typing; –, MLST not determined. †Tick samples (*4*) are indicated by *italics*; strains with genome sequences are indicated in **boldface**. ‡Number of tick extracts with the listed IGS1 locus (numerator)/number of extracts with the listed *ospC* allele (denominator). §MLST from (*4*,*12*) or this study (in parentheses).

## References

[1] Brisson D, Dykhuizen DE. ospC diversity in *Borrelia burgdorferi*: different hosts are different niches. Genetics. 2004;168:713–22. 10.1534/genetics.104.028738 15514047 PMC1448846

[2] Wormser GP, Brisson D, Liveris D, Hanincova K, Sandigursky S, Nowakowski J, *Borrelia burgdorferi* genotype predicts the capacity for hematogenous dissemination during early Lyme disease. J Infect Dis. 2008;198:1358–64. 10.1086/592279 18781866 PMC2776734

[3] Dykhuizen DE, Brisson D, Sandigursky S, Wormser GP, Nowakowski J, Nadelman RB, The propensity of different *Borrelia burgdorferi* sensu stricto genotypes to cause disseminated infections in humans. Am J Trop Med Hyg. 2008;78:806–10. 18458317 PMC2387051

[4] Hoen AG, Margos G, Bent SJ, Diuk-Wasser MA, Barbour AG, Kurtenbach K, Phylogeography *of Borrelia burgdorferi* in the eastern United States reflects multiple independent Lyme disease emergence events. Proc Natl Acad Sci U S A. 2009;106:15013–8. 10.1073/pnas.0903810106 19706476 PMC2727481

[5] Girard YA, Travinsky B, Schotthoefer A, Federova N, Eisen RJ, Eisen L, Population structure of the Lyme disease spirochete *Borrelia burgdorferi* in the western black-legged tick (*Ixodes pacificus*) in northern California. Appl Environ Microbiol. 2009;75:7243–52. 10.1128/AEM.01704-09 19783741 PMC2786521

[6] Qiu WG, Schutzer SE, Bruno JF, Attie O, Xu Y, Dunn JJ, Genetic exchange and plasmid transfers in *Borrelia burgdorferi* sensu stricto revealed by three-way genome comparisons and multilocus sequence typing. Proc Natl Acad Sci U S A. 2004;101:14150–5. 10.1073/pnas.0402745101 15375210 PMC521097

[7] Bunikis J, Garpmo U, Tsao J, Berglund J, Fish D, Barbour AG. Sequence typing reveals extensive strain diversity of the Lyme borreliosis agents *Borrelia burgdorferi* in North America and *Borrelia afzelii* in Europe. Microbiology. 2004;150:1741–55. 10.1099/mic.0.26944-0 15184561

[8] Derdakova M, Beati L, Pet'ko B, Stanko M, Fish D. Genetic variability within *Borrelia burgdorferi* sensu lato genospecies established by PCR-single-strand conformation polymorphism analysis of the *rrfA-rrlB* intergenic spacer in *Ixodes ricinus* ticks from the Czech Republic. Appl Environ Microbiol. 2003;69:509–16. 10.1128/AEM.69.1.509-516.2003 12514035 PMC152394

[9] Qiu WG, Dykhuizen DE, Acosta MS, Luft BJ. Geographic uniformity of the Lyme disease spirochete (*Borrelia burgdorferi*) and its shared history with tick vector (*Ixodes scapularis*) in the northeastern United States. Genetics. 2002;160:833–49. 11901105 10.1093/genetics/160.3.833 PMC1462027

[10] Hanincova K, Liveris D, Sandigursky S, Wormser GP, Schwartz I. *Borrelia burgdorferi* sensu stricto is clonal in patients with early Lyme borreliosis. Appl Environ Microbiol. 2008;74:5008–14. 10.1128/AEM.00479-08 18539816 PMC2519259

[11] Barbour AG, Bunikis J, Travinsky B, Hoen AG, Diuk-Wasser MA, Fish D, Niche partitioning of *Borrelia burgdorferi* and *Borrelia miyamotoi* in the same tick vector and mammalian reservoir species. Am J Trop Med Hyg. 2009;81:1120–31. 10.4269/ajtmh.2009.09-0208 19996447 PMC2841027

[12] Margos G, Gatewood AG, Aanensen DM, Hanincova K, Terekhova D, Vollmer SA, MLST of housekeeping genes captures geographic population structure and suggests a European origin of *Borrelia burgdorferi.* Proc Natl Acad Sci U S A. 2008;105:8730–5. 10.1073/pnas.0800323105 18574151 PMC2435589

[13] Wang IN, Dykhuizen DE, Qiu W, Dunn JJ, Bosler EM, Luft BJ. Genetic diversity of *ospC* in a local population of *Borrelia burgdorferi* sensu stricto. Genetics. 1999;151:15–30. 9872945 10.1093/genetics/151.1.15 PMC1460459

[14] Attie O, Bruno JF, Xu Y, Qiu D, Luft BJ, Qiu WG. Co-evolution of the outer surface protein C gene (*ospC*) and intraspecific lineages of *Borrelia burgdorferi* sensu stricto in the northeastern United States. Infect Genet Evol. 2007;7:1–12. 10.1016/j.meegid.2006.02.008 16684623

[15] Hunter PR, Gaston MA. Numerical index of the discriminatory ability of typing systems: an application of Simpson's index of diversity. J Clin Microbiol. 1988;26:2465–6. 3069867 10.1128/jcm.26.11.2465-2466.1988 PMC266921

